# Transparent Organogels as a Medium for the Light-Induced Conversion from Spiropyran to Merocyanine

**DOI:** 10.3390/gels9120932

**Published:** 2023-11-27

**Authors:** Demetra Giuri, Paolo Ravarino, Claudia Tomasini

**Affiliations:** Department of Chemistry “Giacomo Ciamician”, University of Bologna, Via Piero Gobetti 85, 40129 Bologna, Italy; demetra.giuri2@unibo.it (D.G.); paolo.ravarino@studio.unibo.it (P.R.)

**Keywords:** organogel, self-assembly, low-molecular-weight gelator, peptides, spiropyran, merocyanine, photochromism

## Abstract

Low-molecular-weight peptide gelators are a versatile class of compounds able to form gels under a variety of conditions, even via simple ultrasound sonication. In this paper, the ability of Boc-L-Phe-D-Oxd-L-Phe-OBn to gelate three organic solvents (toluene, tert-butyl methyl ether, and ethanol) was evaluated. The rheological behaviour of the materials was assessed via strain sweep analysis, while the fibrous network was analysed via optical microscopy on the wet gels. The gel obtained from toluene is a highly transparent material, and the one from ethanol appears translucent, while the one from tert-butyl methyl ether is opaque. These gels were used to study the reversible light-induced transformation from spyropiran (SP) to merocyanine (MC) and back, as a model system to check the effect of the gel medium onto the rection kinetic. We observed that the solvent used to form the organogels has a crucial effect on the reaction, as gels from aprotic solvents stabilize the SP form, while the ones from protic solvents stabilize the MC form. We thus obtained a solid support to stabilize the two photochromic species just by changing the solvent polarity. Moreover, we could demonstrate that the self-assembled gels do not interfere with the light-driven conversion process, either starting from SP or MC, thus representing a valid and economical photochromic material.

## 1. Introduction

Low-molecular-weight (LMW) gels are a class of soft materials comprising a fibrous network which entraps a solvent [1,2]. The network is formed by LMW gelators, molecules of reduced size, usually lower than 1000 Da, able to self-assemble through non-covalent interactions, such as hydrogen and halogen bonds [3,4,5,6,7], aromatic π-stackings [8,9], and hydrophobic interactions [10,11].

Peptides are an important class of LMW gelators, because they can self-assemble through a variety of weak interactions, they can be efficiently synthesised, and they facilitate the preparation of materials that are often biocompatible and biodegradable [5,12,13,14,15]. The self-assembly process is usually induced by the application of a trigger, which alters the solubility of the molecules [3,4,5,6,7]. The trigger can be chosen among several physical and chemical inputs (light, temperature, ultrasound sonication, pH, enzymes, salts), and its nature has a huge impact on the final properties of the obtained material [16,17,18,19]. 

The self-assembly of peptide gelators leads to the formation of fibres, usually possessing a β-sheet secondary structure [20,21,22], which entangle together and entrap the solvent. Being composed of weak interactions, these fibres are usually easy to break and reform (reversible gels) [12,23], and the gel formation can be easily tuned [24]. For this reason, LMW gels are useful for several applications, as water decontamination [25,26,27,28,29,30], optical sensors [31,32], template synthesis (crystals, nanostructures) [33,34,35], cell growth [36,37], drug delivery, and release of compounds [38,39,40,41,42]. 

The solvent of these materials can, in turn, be water for hydrogels, or an organic solvent for organogels [43,44,45,46], that can be formed through exposure to ultrasound sonication [11,26] or temperature variation [14,47], often using terminally protected peptides [48,49]. LMW gels are also able to incorporate a variety of species that would not be soluble in the solvent itself. This property can be used to trap photochromic compounds inside a gel, to explore the behaviour of smart materials responding to light in this medium. 

Photochromism is a light-induced phenomenon, which causes the reversible transformation of a species between two forms with different absorption spectra, thus different colours [50,51]. There are many examples of photochromic compounds [52,53,54,55,56], such as azo dyes, spiro-derivatives (spiropyran and spironaphthoxazine), naphthopyrans, quinones, stilbenes, viologens, and diarylethene compounds, possessing different isomers, converting one into the other by changing the wavelength of irradiation. The possibility to transferring the properties of these compounds from solution to solid phase is an interesting topic, as it leads to the preparation of smart materials able to reversibly change optical properties [56,57,58,59]. 

Many photochromic compounds such as spiropyrans [60,61,62,63,64,65,66] were inserted into supramolecular gels, conferring new properties to the functionalized materials. The spiropyran unit (SP) is one of the most studied photochromic compounds [67] for its ability to respond to multiple stimuli, such as light, pH, temperature, and ions, and proved to be a suitable candidate to confer reversible properties to supramolecular gels [68,69,70,71].

**Scheme 1:**
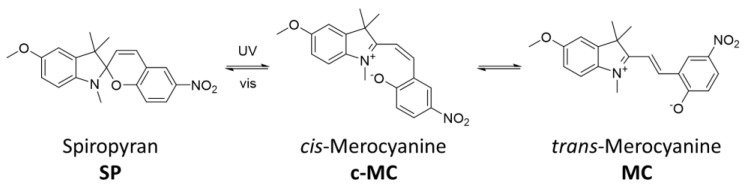
Interconversion between spiropyran, *cis*-merocyanine and *trans*-merocyanine.

In this work, we present the application of a previously reported organogelator [72] that was chosen as it forms transparent organogels with several solvents, thus allowing a large part of visible light to pass through them. We synthesised a SP that can be converted into its merocyanine (MC) form upon UV irradiation (Scheme 1). The reaction occurs through the formation of a *cis*-merocyanine (c-MC), a transient intermediate that isomerises to produce MC when irradiated with UV light, or closes to generate SP when irradiated with visible light. The SP was inserted into the gels prepared with different solvents, and we demonstrated that the SP/MC reaction reversibly occurs both in supramolecular gels and in the solution, being very sensitive to the solvent medium. 

## 2. Results and Discussion

In a previous work from Tomasini et al., the gelator ability of a small panel of molecules containing one or two Phe units has been studied, to check their ability both as hydrogelators and as organogelators [72]. The organogelators are benzyl esters, while the hydrogelators are carboxylic acids. Among these molecules, four contain the L-Phe-D-Oxd-L-Phe (Oxd = 4-carboxy-5-methyl oxazolidin-2-one) unit, in which the two Phe groups are separated by the Oxd group. This choice was dictated by the flourishing results obtained by several research group that used Fmoc-Phe-Phe as gelators [73,74,75,76,77,78,79,80].

**Figure 1:**
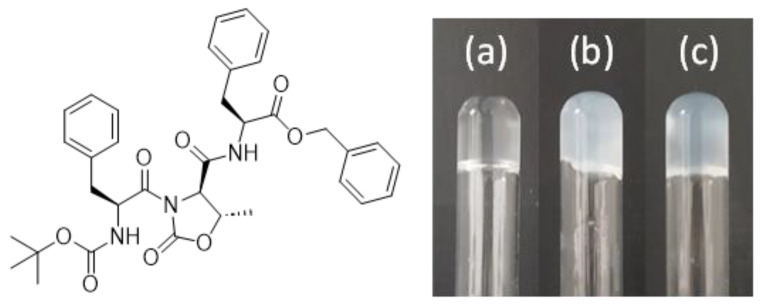
(**Left**) Molecular structure of the gelator Boc-L-Phe-D-Oxd-L-Phe-OBn. (**Right**) Photographs of the organogels obtained from (**a**) toluene, (**b**) TBME, and (**c**) ethanol.

The previously reported molecules differ only for the *N*-protecting group: Fmoc-L-Phe-D-Oxd-L-Phe-OBn, Boc-L-Phe-D-Oxd-L-Phe-OBn, Cbz-L-Phe-D-Oxd-L-Phe-OBn, CH_2_-[(CH_2_)_3_-CO-L-Phe-D-Oxd-L-Phe-OBn]_2_. The gelation ability of these molecules in several organic solvents has been tested. While Fmoc-L-Phe-D-Oxd-L-Phe-OBn and Cbz-L-Phe-D-Oxd-L-Phe-OBn show no ability to form gels under any conditions and CH_2_-[(CH_2_)_3_-CO-L-Phe-D-Oxd-L-Phe-OBn]_2_ gelates only acetonitrile, Boc-L-Phe-D-Oxd-L-Phe-OBn forms strong and transparent organogels in toluene, *tert*-butyl methyl ether (TBME) and ethanol (Figure 1). The rationale behind the gelation behaviour of these molecules was also reported, taking into consideration their hydrophobicity expressed as Log P (octanol/water partition coefficient), calculated as a sum of fragment-based contributions and correction factors. Boc-L-Phe-D-Oxd-L-Phe-OBn has the smallest Log P value (6.489) among all the esters, indicating reduced solubility in organic solvents, an important parameter for the design of new LMWGs.

Due to the transparency observed for these there organogels, we now choose these materials as perfect media to study the light-induced conversion from spiropyran to merocyanine. Moreover, the three solvents differ with respect to their properties as toluene and TBME are aprotic solvents, while ethanol is a protic solvent, so they may affect differently the kinetic of this conversion.

The three organogels have been previously characterized via the analysis of the Tgel and the SEM study of the corresponding xerogels, but neither studies on the rheological properties nor studies on the minimum gelation concentration (MGC) had been performed, so we performed these measurements first. 

The minimum gelation concentration (MGC) of the gels was evaluated for the three solvents and the result was 0.3% *w*/*v* for toluene and TBME, and 0.5% *w*/*v* for EtOH (Figure S1). Since the aim of the study is to report a material behaving as a solid and robust support for the light-driven conversion of a photochromic compound, the concentration of the gelator was increased to 1% *w*/*v* for all the following studies, to obtain a stiffer material, which also preserved the transparency.

All the gels were prepared at 1% *w*/*v* gelator concentration, sonicated at room temperature for 20 min and left to rest overnight for the complete gel formation.

**Figure 2:**
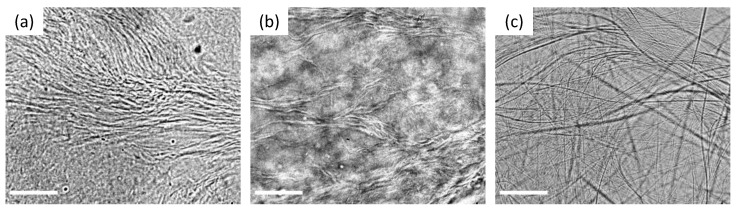
Optical microscope images of the organogels obtained from (**a**) toluene, (**b**) TBME, and (**c**) ethanol. The scalebar is 50 µm.

As previously observed, the gels obtained from toluene and ethanol appear translucent, while the one from TBME is opaque, but still disclosing a good transparency (Figure 1). The fibrous network of the gels was already studied via the analysis of the corresponding xerogel [72], but this analysis is always affected by the lack of the solvent, so now we analysed the wet organogel via optical microscopy with a 40× magnification (Figure 2). In all the cases, a network of thin long fibres was revealed (as in the SEM analysis of the corresponding xerogels). In toluene and TBME, fibres appear in bundles, while in ethanol, they are isolated.

**Figure 3:**
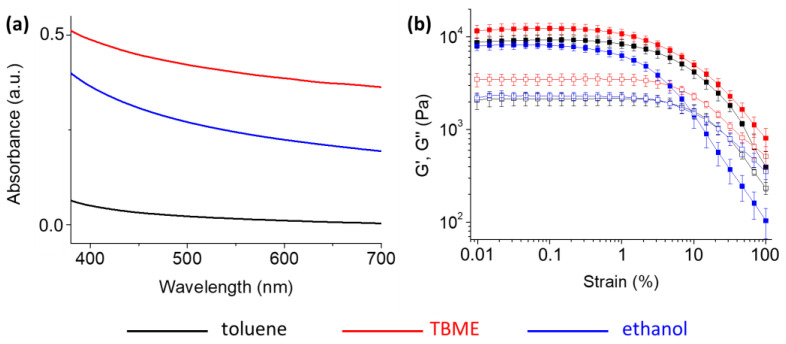
(**a**) Normalised absorption spectra (380–700 nm) and (**b**) strain sweep experiments of the gels prepared in in toluene (black), TBME (red), and ethanol (blue) in 1% *w*/*v* concentration. The solid symbols represent G′, the open ones G″.

The organogels transparency was probed by measuring the absorbance of the gels prepared in quartz cuvettes with a path length of 5 mm in the visible region (380–700 nm) (Figure 3a). The spectra showed a broad range of transparency. We calculated the transparency at 630 nm [6], obtaining a transmittance of 99.2% for toluene, 42.0% for TBME, and 61.1% for ethanol. The value at 630 nm was chosen as the middle value in the range 560–700 nm where the gels showed the highest transparency (Table S1).

Then, we studied the rheological behaviour of the materials via strain sweep analysis using the cup and vane geometry (Figure S1). The three gels display a stiffness of ranging between 14,500 and 9200 Pa (Figure 3b), with the one in TBME being the stiffest. As we previously reported [6], it is not unusual that a reduced transparency corresponds to an increased stiffness. The gels in toluene and TBME also display a long linear viscoelastic range, and the moduli G′ and G″ do not show a crossover point, meaning that the gels do not break in the studied strain range.

As a possible application of the transparent supramolecular material, we prepared six supramolecular gels in toluene, TBME, and ethanol, all containing either SP or MC in the three solvents. The SP compound chosen (Scheme 1) was synthesised following a previously reported procedure, and the characterization matched values in the literature [81]. All the gels contain the gelator in 1% *w*/*v* ratio, and either of the two molecules (SP or MC) in 0.5% *w*/*v* ratio. The formation of all these functionalized organogels was not hampered by the presence of the two light-responsive compounds. 

**Figure 4:**
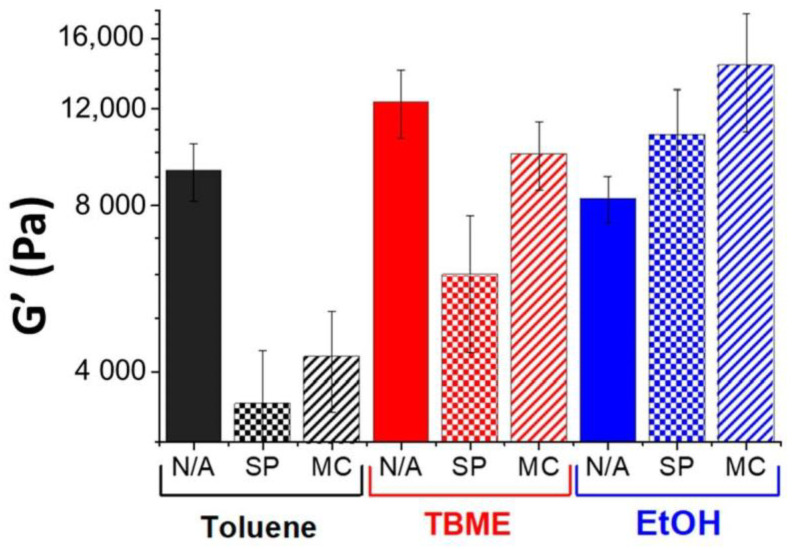
Comparison among the stiffness with error bars of the organogels (taken from Figure S2, γ = 0.05%) obtained from toluene (black), TBME (red), and ethanol (blue). Samples labelled with N/A contain only the gelator, samples labelled with SP contain the gelator and SP, and samples labelled with MC contain the gelator and MC.

The properties of the new gels were studied by strain sweep analysis, under the above reported conditions (Figure S2). As depicted in the graph reported in Figure 4, organogels of toluene and TBME containing either the SP or the MC are weaker compared to the gels containing only the gelator, while the opposite phenomenon occurs in the case of ethanol. However, the differences are small, as the stiffness is in the same order of magnitude of the gels without either of the molecules, ranging between 3.52 and 12.3 kPa. 

Another piece of information necessary to study the formation of the fibres which constitute the gel is obtained via the analysis of the IR spectra of the wet organogels prepared in 1% *w*/*w* concentration (Figure S4). The stretching bands of NH groups in the aprotic solvents (toluene and TBME) are located at 3339 and 3336 cm^−1^, respectively. This is the typical position of the NH groups that are involved in hydrogen bonding, requested to form the supramolecular fibres. Unfortunately, the NH stretching band in the ethanol organogel is not visible as it is covered by the overwhelming signal of the OH group.

Then, the effect of the three media on the equilibrium among SP and MC was studied both in the solutions and in the gels. First, we prepared three solutions of the product in the three solvents in 5 mg/mL concentration. The solutions in toluene and TBME are yellow, indicating that the equilibrium in these solvents privileged the SP form. In contrast, the ethanol solution is red, suggesting that in this case the MC form is preferred [82]. These results agree with the different polarity of the solvents: aprotic solvents stabilise the SP structure, while the protic solvent stabilises the zwitterionic MC form.

**Figure 5:**
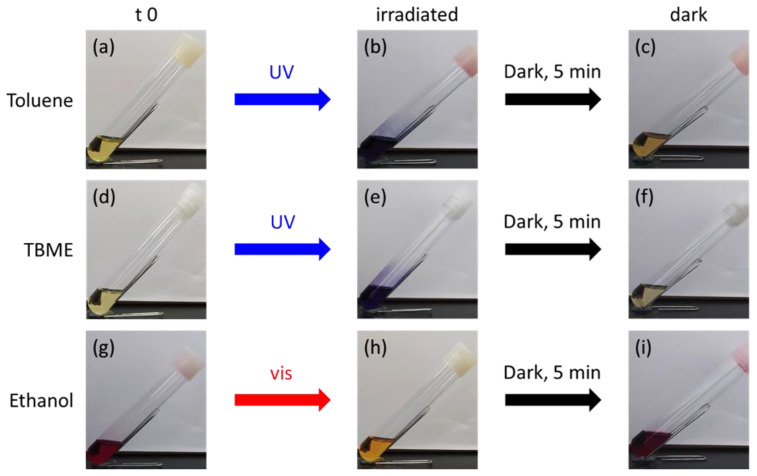
Photographs of the SP solutions in (**a**–**c**) toluene, (**d**–**f**) TBME, and (**g**–**i**) ethanol, after (**a**,**d**,**g**) dissolution of SP, (**b**,**e**,**h**) irradiation, and (**c**,**f**,**i**) a rest of 5 min while wrapped in aluminium foil. For (**b**,**e**), UV light (365 nm) was used, while for (**h**), white light was implied. In all cases, the concentration of SP and MC is 0.5% *w*/*v*.

To verify that the light-driven interconversion would occur in any solvent, the solutions in toluene and TBME were irradiated with UV light (365 nm) to allow the complete conversion to MC, with the formation of a blue solution. We repeated the same process for the ethanol solution containing MC, irradiating it with white light to induce the formation of SP (Figure 5).

After irradiation, each solution was wrapped in aluminium foil to mimic a completely dark environment and left to rest 5 min. After this time, each solution reconverted to the starting point (Figure 5), thus confirming that the protic solvents stabilize MC, while aprotic solvents stabilize SP.

To check if the solvent stabilization effect prevails also in organogels, the experiment was repeated by including SP or MC in the three gels, preparing two samples of each organogel. For this purpose, the gelator (1% *w*/*v* concentration) and SP (5 *w*/*v* concentration) were dissolved in the three solvents. Then, one group of the three gels was irradiated with UV light and the other group with white light during the whole process of sonication, then the gels were wrapped in aluminium foil to mimic dark conditions and left to rest overnight. The additional presence of the photochromic species did not hamper the organogel formation. 

**Figure 6:**
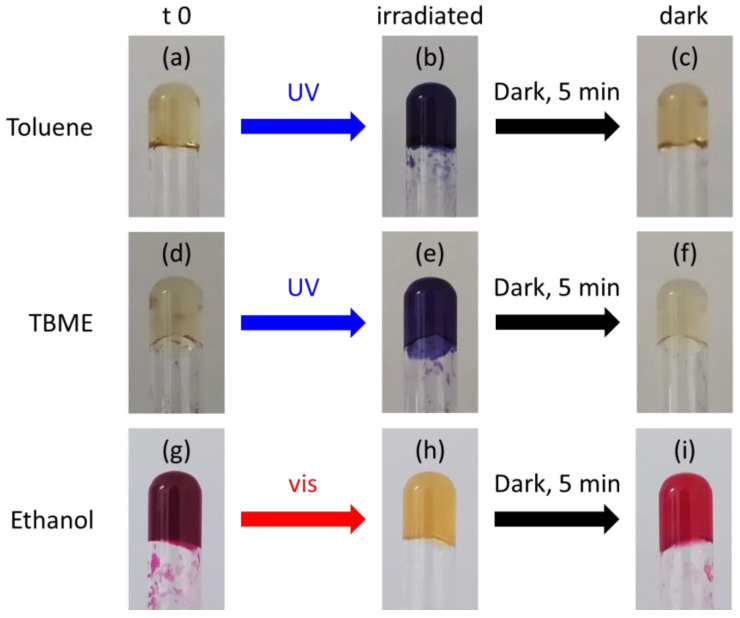
Photographs of the organogels in (**a**–**c**) toluene, (**d**–**f**) TBME, and (**g**–**i**) ethanol, after (**a**,**d**,**g**) an overnight rest in the dark, (**b**,**e**,**h**) irradiation with (**b**,**e**) UV (365 nm) or (**h**) white light, and (**c**,**f**,**i**) after 5 min in the dark. In all cases, the concentration of gelator is 1% *w*/*v* and that of SP is 0.5% *w*/*v*.

On inspection after this time, gels in toluene and in TBME from both groups were yellow, indicating that the SP form prevails. These gels were irradiated again with UV light after complete formation, wrapped in aluminium foil and checked after 5 min. After this time, the conversion to SP was complete (Figure 6). 

In contrast, after this time, the organogels in ethanol from both sets were red, suggesting that the MC form prevails as in the solution (Figure 6). The two gels were irradiated with visible light after their complete formation, wrapped in aluminium foil, and checked after 5 min. Within this time, the reconversion to MC took place.

To have a better understanding of this phenomenon, a spectrophotometric study was carried out. To record the UV-vis absorption spectra, we prepared diluted solutions containing 0.05 mg/mL of either SP or MC, as at higher concentrations (5 mg/mL) the signal is too intense.

We prepared six solutions, three of which containing SP (group A’) and the other three containing MC (group B’) in the three solvents, and recorded the UV-vis spectra. Group A’ was irradiated with visible light for 1 min to allow the complete formation of SP, while group B’ was irradiated with UV light for 1 min to allow the complete formation of MC. We repeated the same set of measurements on the six solutions that had been jellified, adding to each of them the gelator in 1% *w*/*v* concentration, and recorded the UV-vis spectra.

**Figure 7:**
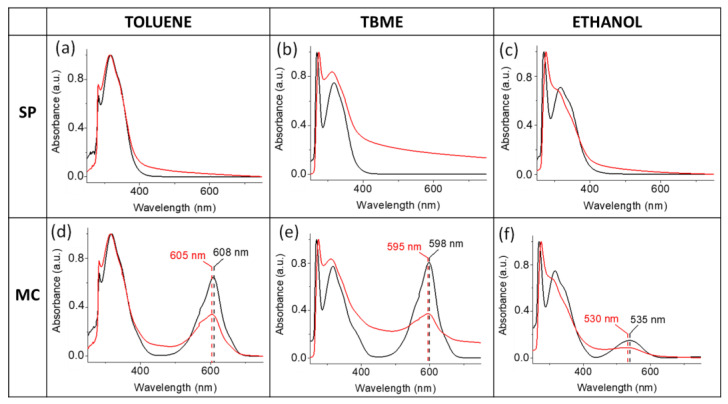
UV-vis absorption spectra of 0.05 mg/mL solutions (black) and gels (red) of SP (**a**–**c**) or MC (**d**–**f**) in toluene (**a**,**d**), TBME (**b**,**e**), and ethanol (**c**,**f**). To obtain SP, the solutions were irradiated for 1 min with white light, while to obtain MC, the solutions were irradiated for 1 min with 365 nm UV light. Gels were formed in the mentioned solutions after 5 min of sonication.

The results are summarized in Figure 7. In each illustration, we compared the signals of the same solution of SP or MC before and after jellification. The spectra are overall very similar, although we can notice small differences in Figure 7d–f. Indeed, the peaks that can be ascribed to MC (at 608, 598, and 535 nm, respectively) are always much more intense in the organogels, together with a small blue-shift (3–5 nm) from the maxima in solution to the maxima in gel. Unfortunately, we could not compare the UV-vis absorption spectra of the SP form in the gel and solution because the signal of the gelator covered the one of the SPs in the region below 400 nm (Figure 7a–c).

**Figure 8:**
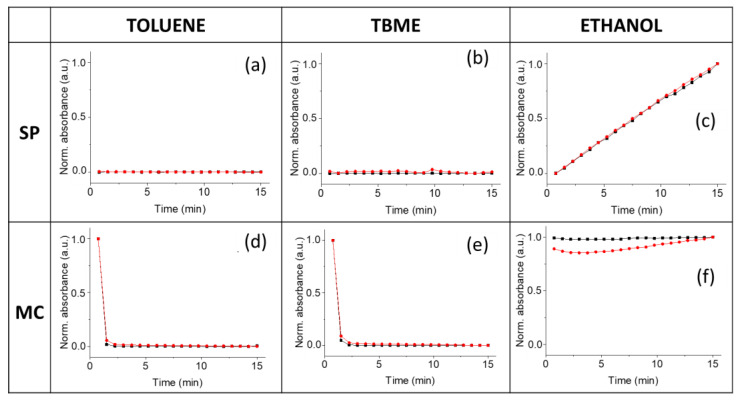
Trend of the normalised absorbance versus time of the solution (black) and gel (red) at the maximum corresponding to the MC signal. Media contain either (**a**–**c**) SP or (**d**–**f**) MC. The solvents are (**a**,**d**) toluene, (**b**,**e**) TBME, and (**c**,**f**) ethanol. In each case, the concentration of SP or MC is 0.005% *w*/*v*.

Finally, the UV-vis absorption of the twelve samples (before and after jellification) was recorded over 15 min (Figures S5 and S6). For each cycle, the intensity of the MC signal was recorded, and its maximum absorbance (608 nm for toluene, 598 nm for TBME, and 535 nm for ethanol, respectively) was measured and compared (Figure 8). 

Gels of toluene and TBME stabilise the SP form (Figure 8d,e), while gels of ethanol convert the SP into the MC form (Figure 8c), thus confirming that the solvent is the principal parameter that affects the equilibrium between the two forms, as previously observed. 

## 3. Conclusions

In this work, we prepared three organogels obtained from organic solvents of different polarity (toluene, TBME, and ethanol) and the gelator Boc-L-Phe-D-Oxd-L-Phe-OBn in 1% *w*/*v* concentration. The organogels were characterized in terms of rheological, morphological, and optical properties. These materials are highly transparent, and their transparency increases from TMBE, to ethanol to toluene, the last being highly transparent (transmittance of 99.2% at 630 nm). To apply this remarkable property, the gels were used to study the light-catalysed conversion between spiropyran and merocyanine, which are photochromic compounds.

The presence of these light-sensitive species has no impact on the gelation ability of the gelator and a reduced impact on the final rheological properties of the materials, leaving mostly unaltered their stiffness, breaking point, and linear viscoelastic range. 

Moreover, the photochromic compounds always behave in the gel matrix as in solution, interconverting to the most stable form, that is, SP for aprotic solvents, and MC for protic solvents. Finally, the interconversion follows the same kinetics both in the gel and in the solution, as the use of a transparent gel does not interfere with the medium irradiation, both with UV and with visible light. 

We demonstrated that the organogels presented in this work are transparent smart materials that are good candidates to host photochromic compounds, as they never hinder the reversible interconversion between the SP and MC forms, while acting as solid and economic supports.

## 4. Materials and Methods

Gelator Synthesis. The molecule was prepared via coupling of Boc-L-Phe-D-Oxd-OH [1] and H-Phe-OBn using the solution phase technique. Characterization: M.p. = 196–198 °C; [α]_D_^20^ 31.1 (c = 0.1 in CH_2_Cl_2_); IR (CH_2_Cl_2_, 3 mM) ν = 3432, 3350, 1788, 1740, 1700, 1684 cm^−1^; ^1^H NMR (400 MHz, CDCl_3_): δ 1.26 (d, *J* = 6.4 Hz, 3H, Me-Oxd), 1.33 (s, 9H, O-*t*Bu), 2.80–2.92 (m, 1H, CHβ-Phe), 3.01–3.21 (m, 3H, CHβ-Phe + CH_2_β-Phe), 4.21 (bs, 1H, CHN-Oxd), 4.53 (dq, *J* = 5.2, 6.4 Hz, CHO-Oxd), 4.75 (q, *J* = 6.8 Hz, CHα-Phe), 4.98–5.17 (m, 3H, NH + OCH_2_Ph), 5.59 (bs, 1H, CHα-Phe), 6.94–7.44 (m, 16 H, 15 Ar + 1 NH); ^13^C NMR (100 MHz, CDCl_3_): 20.7, 28.2, 37.3, 54.1, 62.6, 67.2, 74.7, 80.5, 127.2, 128.5, 128.7, 129.2, 129.4, 135.2, 135.5, 136.0, 151.5, 166.9, 170.5, 172.8. Elemental analysis calcd (%) for C_35_H_39_N_3_O_8_: C, 66.76; H, 6.24; N, 6.67; found: C, 66.80; H, 6.28; N, 6.71.

Formation of gel. Gels of Boc-L-Phe-D-Oxd-L-Phe-OBn were prepared in 1% *w*/*v* concentration (10 mg/mL) of gelator in a glass test tube (diameter = 16 mm) with fresh solvents in a total volume of 2.5 mL. The mixture was sonicated for 20 min until a homogeneous phase was obtained, and then the samples were left to rest overnight to allow complete gel formation.

Formation of Gels containing SP or MC. SP and MC solutions in 5 mg/mL concentration were freshly prepared by swirling the solid in toluene, TBME or ethanol. SP and MC solutions in 0.05 mg/mL concentration were prepared via dilution of the 5 mg/mL solutions with fresh solvent.

Gels containing SP or MC for rheological analysis were prepared with the gelator in 1% *w*/*v* concentration (10 mg/mL) and either SP or MC in 0.5% *w*/*v* concentration (5 mg/mL) in a total volume of 2.5 mL. The mixture was sonicated for 20 min until a homogeneous phase was obtained, then the samples were left to rest overnight to allow complete gel formation.

Gels containing SP or MC for spectrophotometric analysis were prepared with the gelator in 1% *w*/*v* concentration (10 mg/mL) and either SP or MC in 0.005% *w*/*v* concentration (0.05 mg/mL) in a total volume of 1.5 mL. The mixture was sonicated for 15 min until a homogeneous phase was obtained, and then it was pipetted in a quartz cuvette (path length 5.0 mm), sonicated for additional 5 min, and left to rest overnight to allow complete gel formation.

Gels containing SP were prepared by pouring the solution of SP irradiated with white light (300 W) to ensure complete conversion, on the solid gelator. Then, the solutions were sonicated maintaining the visible irradiation with a LED lamp (2 W), and as they were removed from the sonicating bath, they were wrapped in aluminium foil to mimic dark conditions and left to rest overnight. 

Gels containing MC were prepared in the same way, replacing the use of white light with the use of UV light (365 nm, 10 W).

All gels were prepared using an Elma (Singen, Germany) Elmasonic S 30 H ultrasound sonicator (37 kHz).

Optical microscopy. The images were recorded using a Nikon (Tokyo, Japan) A1R optical microscope and a 40× magnifier. Gels used for optical microscopy were prepared as described, and then a small piece of the gel was transferred onto a glass microscope slide and gently covered with a cover slip.

Rheology. All rheological measurements were performed using an Anton Paar (Graz, Austria) MCR102 rheometer. A vane and cup measuring system was used, setting a gap of 2.1 mm. The gels were prepared as described and tested directly in the glass test tube (diameter = 16 mm). Oscillatory amplitude sweep experiments (γ: 0.01–100%) were performed at a fixed temperature of 23 °C, controlled using an integrated Peltier system, using a constant angular frequency (ω) of 10 rad/s. All analyses were performed after 16 h from the removal of the gel solution from the sonication bath, to allow a complete gel formation, and repeated in triplicate.

Spectrophotometric characterization. Gels used for probing the transparency of the gels media were prepared as described, by using 1.5 mL of the homogeneous phase containing 1.0% *w*/*v* of **1** in the solvent chosen. Then, this amount was transferred in a quartz cuvette (path length 5.0 mm) during sonication before gelation occurred, and left to rest overnight to allow complete gel formation. 

Solutions of SP and MC were prepared by dissolving 0.05 mg/mL of SP in the required solvent and irradiating it with either white (300 W) or UV (365 nm) light, respectively, for 1 min to ensure complete conversion to the needed specimen.

Gels used for studying the conversion from SP to MC, and vice versa, were prepared in the same way, replacing the fresh solvent with a 0.05 mg/mL solution of either SP or MC in the required solvent. The SP solution was obtained after irradiation with white light (300 W), and the MC solution was obtained after irradiation with UV light (365 nm).

## Data Availability

The authors confirm that the data supporting the findings of this study are available within the article and its Supplementary Materials.

## Supplementary Materials

The following supporting information can be downloaded at: https://www.mdpi.com/article/10.3390/gels9120932/s1, Figure S1: Photograph of the MGC study for the three solvents; Table S1: Transparency calculation starting from the spectrophotometric analysis in Figure 3a; Figure S2: Photograph of the cup and vane geometry adopted for the rheological experiments; Figure S3: Strain sweep experiments of the gels in toluene, TBME, and ethanol, each containing SP or MC in 0.5 *w*/*v* concentration; Figure S4: FT-IR spectra of the organogels obtained in 1% *w*/*v* concentration in toluene (top), TBME (middle) and ethanol (bottom); Figure S5: Normalised UV-vis absorption spectra over time of solutions and gels containing SP in 0.005 *w*/*v* concentration; Figure S6: Normalised UV-vis absorption spectra over time of solutions and gels containing MC in 0.005 *w*/*v* concentration.
