# Supplementary material for: Transparent Organogels as a Medium for the Light-Induced Conversion from Spiropyran to Merocyanine
Source: Gels. 2023 Nov 27;9(12):932. doi: 10.3390/gels9120932 (PMC10742928; doi:10.3390/gels9120932)
Supplement: Supplementary file 1 [file gels-09-00932-s001.zip › gels-2721342-supplementary.pdf]

SUPPORTING INFORMATION

Transparent Organogels as a Medium for the Light-Induced Conversion from Spiropyran to Merocyanine

Demetra Giuri, Paolo Ravarino and Claudia Tomasini*

[a] *Department of Chemistry "Giacomo Ciamician", University of Bologna, Via Piero Gobetti, 85 - 40129 Bologna - Italy*

**Figure S1.** Photograph of the MGC study for the three solvents.	Page S2
**Table S1.** Transparency calculation starting from the spectrophotometric analysis in Figure 3a	Page S3
**Figure S2.** Photograph of the cup and vane geometry adopted for the rheological experiments.	Page S3
**Figure S3.** Strain sweep experiments of the gels in toluene, TBME, and ethanol, each containing SP or MC in 0.5 w/V concentration.	Page S4
**Figure S4.** FT-IR spectra of the organogels obtained in 1% w/V concentration in toluene (top), TBME (middle) and ethanol (bottom).	Page S5
**Figure S5.** Normalised UV-vis absorption spectra over time of solutions and gels containing **SP** in 0.005 w/V concentration.	Page S6
**Figure S6.** Normalised UV-vis absorption spectra over time of solutions and gels containing **MC** in 0.005 w/V concentration.	Page S6

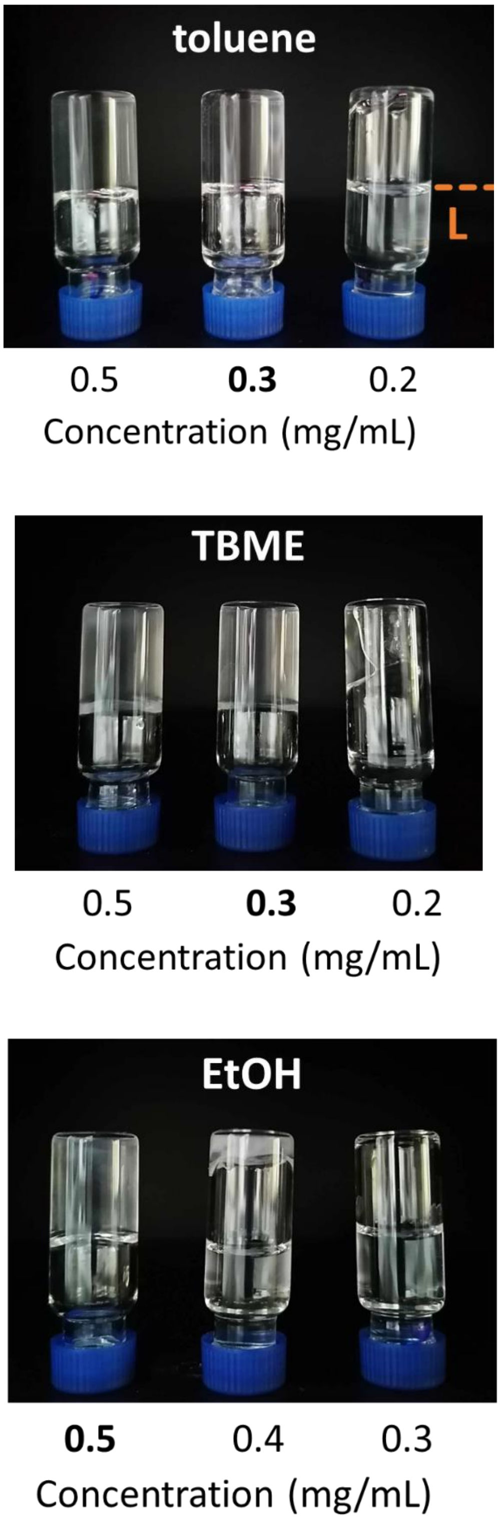

Figure S1. Photographs of the MGC study for the three solvents: toluene, TBME and EtOH.

Table S1. Transparency calculation starting from the spectrophotometric analysis in Figure 3a.

Solvent	A (λ=630 nm)	T (%)
Toluene	0.078906	99.2
TBME	0.37697	42.0
EtOH	0.21375	61.1

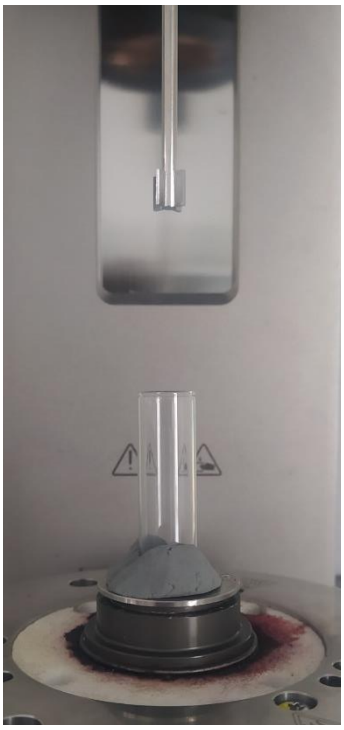

Figure S2. Photograph of the cup and vane geometry adopted for the rheological experiments. Sterilin cups were replaced with 16 mm diameter glass test tube.

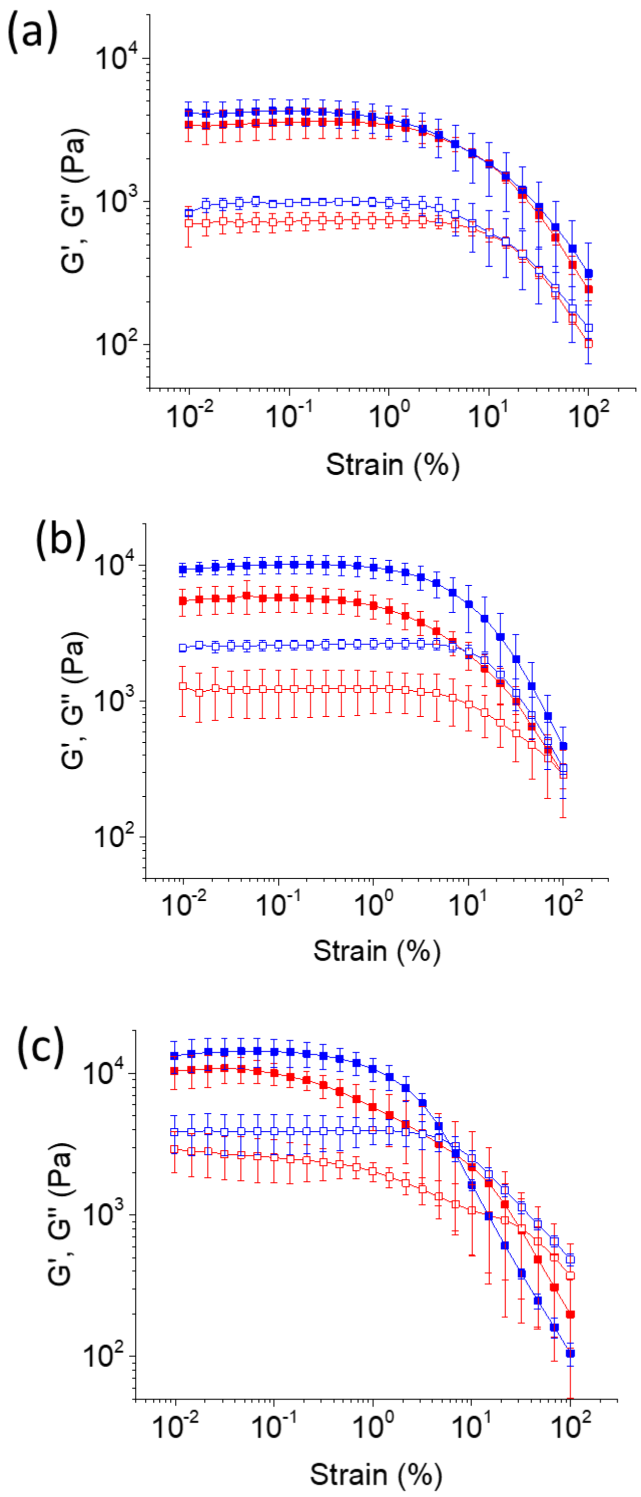

Figure S3. Strain sweep experiments of the gels in (a) toluene, (b) TBME, and (c) ethanol each containing SP (red symbols) or MC (blue symbols). Solid symbols represent G’, open symbols represent G’’.

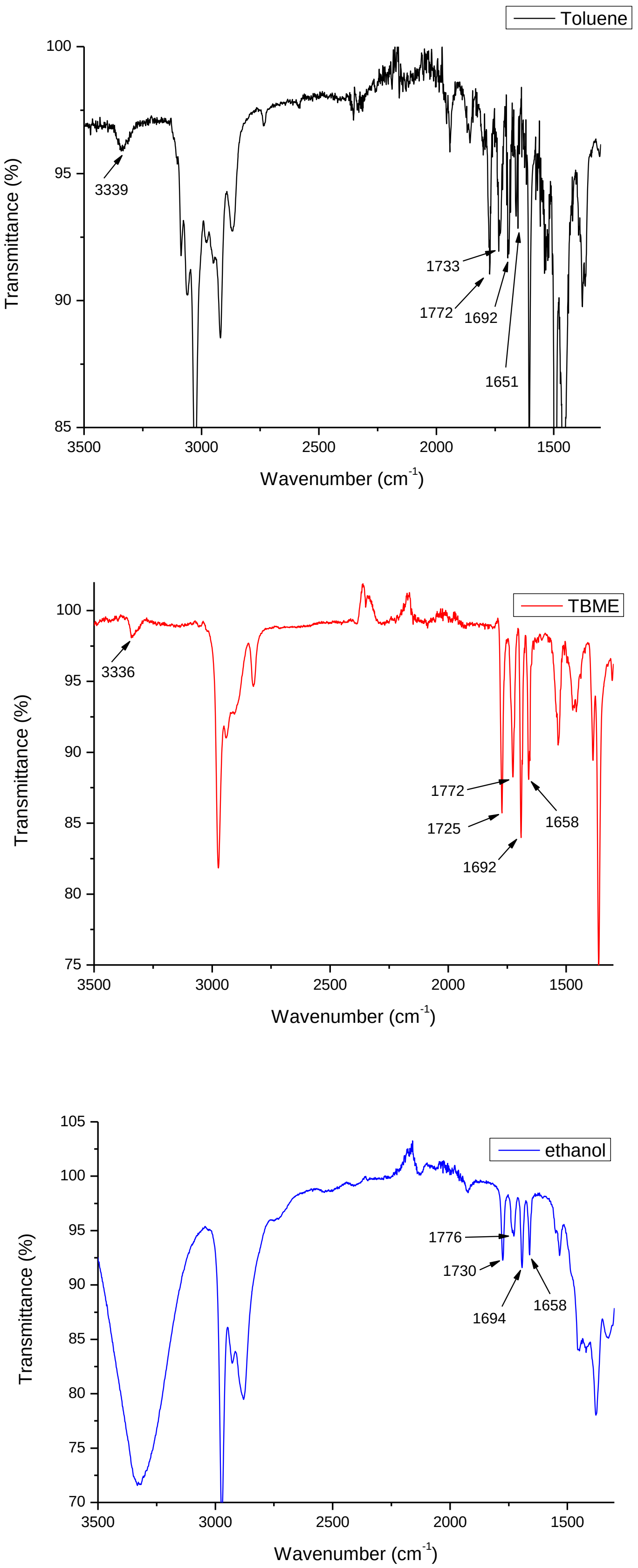

Figure S4. FT-IR spectra of the organogels obtained in 1% w/V concentration in toluene (top), TBME (middle) and ethanol (bottom).

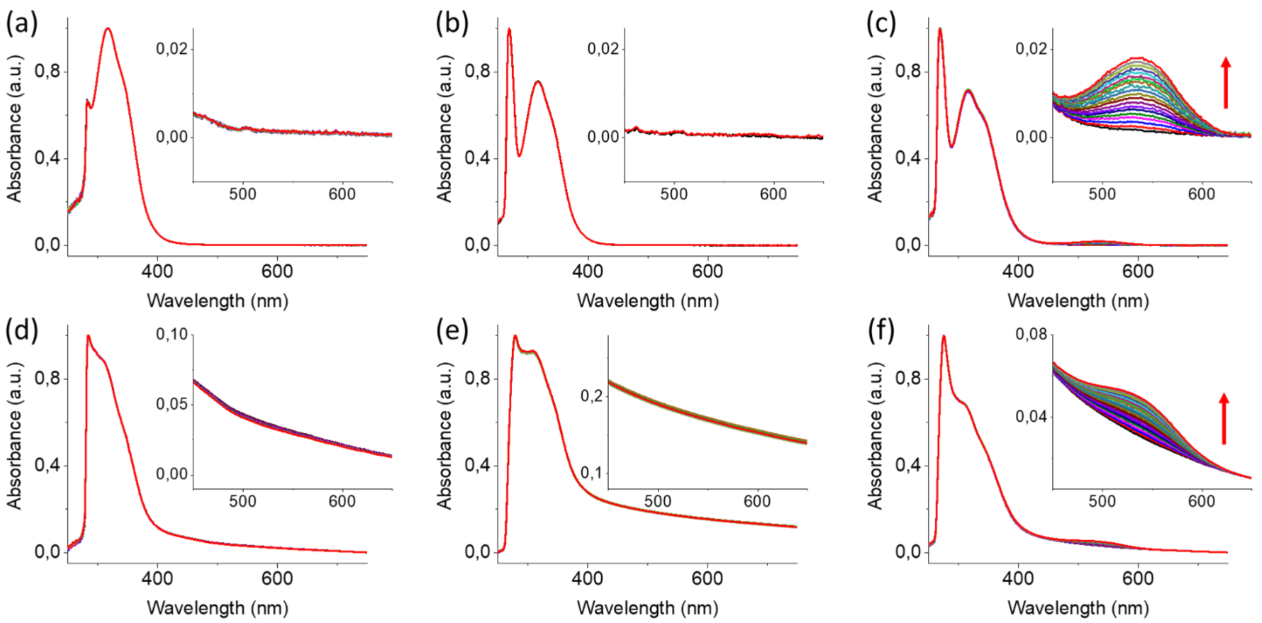

Figure S5. Normalised UV-vis absorption spectra over time of (a-c) solutions and (d-f) gels containing **SP** (0.05 mg/mL) obtained from (a, d) toluene, (b, e) TBME, and (c, f) ethanol in the range 250-750 nm, with zoom in the region 450-650 nm. Red arrows are meant to underline the trend of the absorption variation. Gels are made with 10 mg/mL of the gelator.

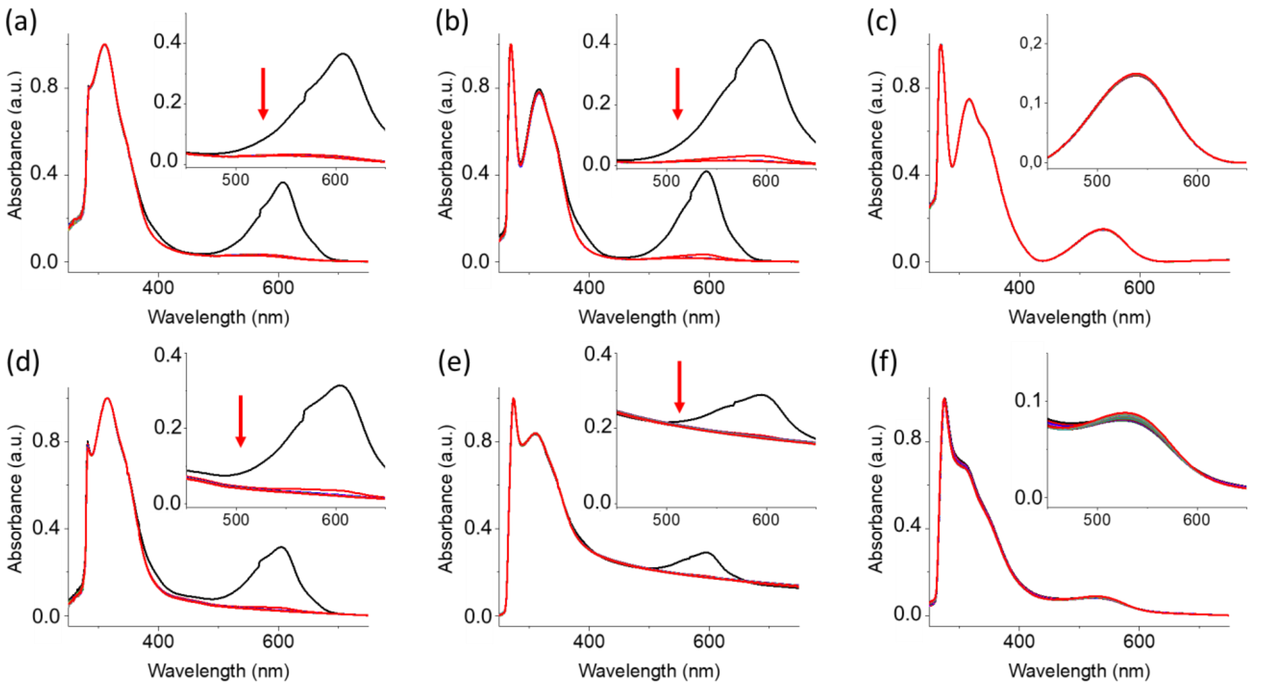

Figure S6. Normalised UV-vis absorption spectra over time of (a-c) solutions and (d-f) gels containing 0.05 mg/mL of **MC** obtained from (a, d) toluene, (b, e) TBME, and (c, f) ethanol in the range 250-750 nm, with zoom in the region 450-650 nm. Red arrows are meant to underline the trend of the absorption variation. Gels are made with 10 mg/mL of the gelator.
